# Enantioselective, Organocatalytic Morita-Baylis-Hillman and Aza-Morita-Baylis-Hillman Reactions: Stereochemical Issues

**DOI:** 10.3390/molecules15020709

**Published:** 2010-02-01

**Authors:** Javier Mansilla, José M. Saá

**Affiliations:** Departamento de Química, Universidad de las Islas Baleares, 07122, Palma de Mallorca, Spain

**Keywords:** Morita-Baylis-Hillman reaction, aza-Morita-Baylis-Hillman reaction, stereochemistry, bifunctional catalysts, polyfunctional catalysts, (*S*)-proline-amine co-catalysis

## Abstract

Conscious of the importance that stereochemical issues may have on the design of efficient organocatalyts for both Morita-Baylis-Hillman and aza-Morita-Baylis-Hillman reaction we have analyzed them in this minireview. The so-called standard reactions involve “naked” enolates which therefore should lead to the *syn* adducts as the major products, irrespective of the *E, Z* stereochemistry of the enolate. Accordingly, provided the second step is rate determining step, the design of successful bifunctional or polyfunctional catalysts has to consider the geometrical requirements imposed by the transition structures of the second step of these reactions. On the other hand, MBH and aza-MBH reactions co-catalyzed by (*S*)-proline and a secondary or tertiary amine (co-catalyst) involve the aldol-type condensation of either a 3-amino-substituted enamine, dienamine, or both, depending on the cases. A Zimmerman-Traxler mechanism defines the stereochemical issues regarding these co-catalyzed condensations which parallel those of the well established (*S*)-proline catalyzed aldol-like reactions.

## 1. Introduction

**Scheme 1:**
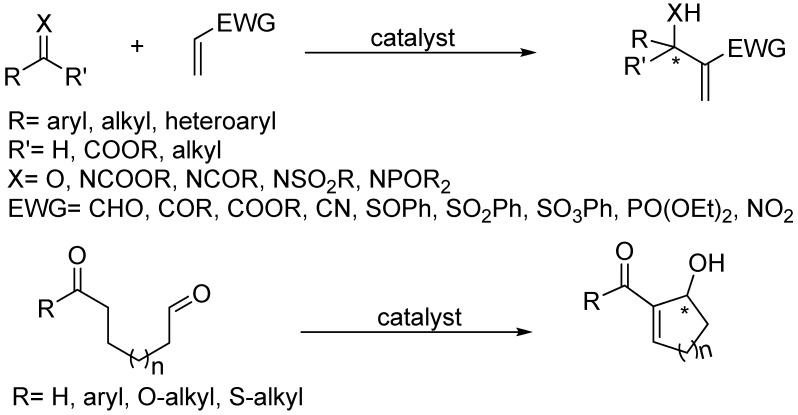
Morita-Baylis-Hillman (MBH) and aza-Morita-Baylis-Hilman (aza-MBH) reactions.

The original Morita-Baylis-Hillman (MBH) reaction [1,2] and its aza analogue (aza-MBH) [3] are unique reactions in many respects, the most relevant being perhaps its atom-economic and organocatalytic nature [4,5,6,7,8,9,10]. The standard reactions typically require bulky, conformationally rigid, basic tertiary amines such as quinuclidines [11,12,13], though DBU [14], DMAP [15], imidazoles [16], guanidine [17], or even heterocyclic carbenes [18,19] or nucleophilic phosphines [20,21] acting as Lewis base catalysts [22] have been used as well for promoting the condensation of an؟ α,β-unsaturated systems (aldehydes, ketones, esters, nitriles, amides, phosphonates, sulphonates, sulfones, sulfoxides or nitro compounds have been employed) with either the C=O functionality present in aldehydes, ketones or α-keto esters for the case of MBH reactions, or with the C=N moiety of *N*-sulfonyl, *N*-acyl, *N*-phosphinoyl, and *N*-alkoxycarbonyl imines in the case of aza-MBH reactions. The products are densely functionalized small molecules whose basic skeleton is that of a chiral, cyclic, or acyclic, α-methylene-β-hydroxycarbonyl, or α-methylene-β-aminocarbonyl, compound for MBH [23,24,25,26,27,28,29,30,31], or aza-MBH reactions [32,33,34,35], respectively. Both skeletons have attracted great synthetic interest, especially when derived from prochiral C=O or C=N funcionalities since enantioselective versions could then be designed (Scheme 1). 

Needless to say, great efforts have been made by researchers all over the world to find efficient catalysts for achieving highly enantioselective MBH and aza-MBH reactions. The challenge was and still is huge, as investigators need to overcome many reaction hurdles such as low conversions, long reaction times and poor enantioselection. Impulse for the research race has come from extensive mechanistic studies.

**Scheme 2:**
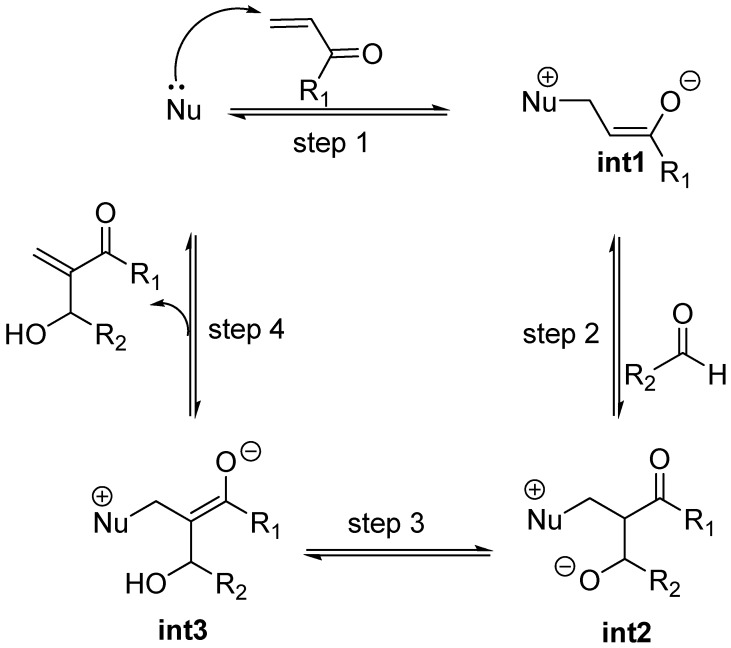
Original mechanistic proposal for the MBH reaction.

The original mechanism for the standard MBH reaction cycle, proposed by Hill and Isaacs [36,37], invoked a series of four transition structures giving rise to three highly dipolar, zwitterionic intermediates eventually collapsing to the final adduct together with the catalyst, then ready for a new cycle (Scheme 2). Strong support for the original MBH mechanism has come in the past from kinetic studies [38,39,40,41], and most recently by NMR studies [42] and ESI-MS data [43], according to which the aldol addition step (step 2 in Scheme 2) should be the rate determining step. Being highly dipolar means that the zwitterionic intermediates, as well as their preceding transition structures, should be high energy species impossible to detect or isolate in most cases [44], even if a specific, internal stabilization could be provided. Actually, it was soon recognized that Brönsted acid additives (water, methanol, ureas, thioureas, *etc.*) accelerated MBH reactions [45,46,47,48,49,50,51,15], thus spurring the search for suitable chiral, bifuntional molecules having a Lewis base (usually a rigid, tertiary amine or phosphine) and a Brönsted acid appropriately located for stabilizing those zwitterionic species and their precursory transition structures, *i.e.,* LBBA catalysts [52,53,54,55,56,57,58,42].

**Scheme 3:**
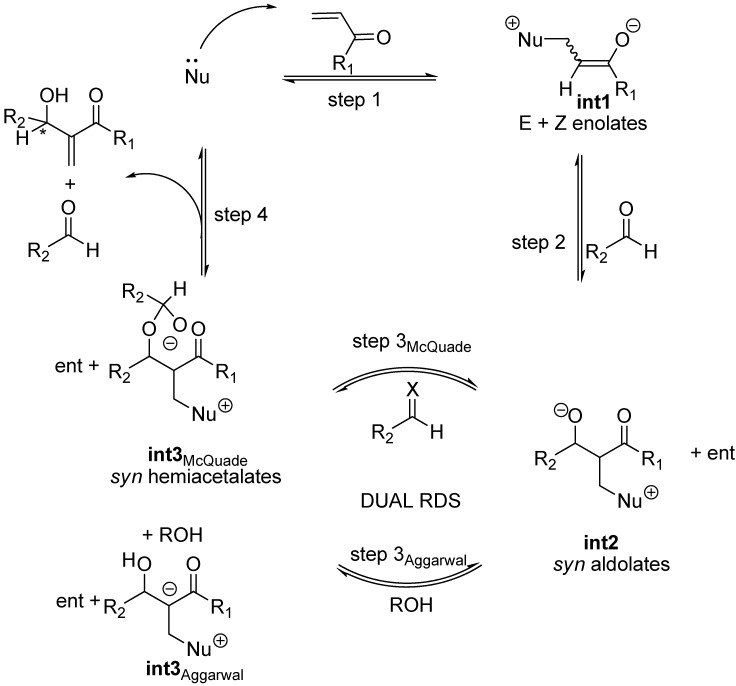
Dual mechanism for the standard MBH reactions according to recent physico-chemical studies.

Recent experimental results and theoretical studies regarding the third step of the standard MBH and aza-MBH reactions (step 3 in Scheme 2) suggested a dualistic nature for it, thus significantly modifying the original mechanism. On the one hand McQuade has presented two key observations for quinuclidine-catalyzed MBH reaction of acrylate and benzaldehyde catalyzed by DABCO in non-polar, polar or even protic solvents, namely: 1) the rate equation is first order in DABCO and acrylate and second order in aldehyde, and 2) a large kinetic isotope effect was observed when α-deuterioacrylate was employed [59,60]. Altogether these facts are strong support for McQuade’s claim for a new mechanism for the standard MBH racemic reaction, according to which the rate determining step must be the proton shift occurring in the third step upon a hemiacetalate species. On the other hand, kinetic studies by Aggarwal *et al*. showed that the standard MBH reaction (ethyl acrylate with benzaldehyde catalyzed by quinuclidine) is autocatalytic at short reaction times, the implications being that in the presence of a proton donor molecule (alcohol, water, *etc.*) the rate determining step should be the protonation of the intermediate ammonium aldolate (**int2**) with the concomitant removal of the carbonyl α-hydrogen [61]. This proposal was further supported by computational data which showed that the energy barrier for this ROH-promoted proton shift was even somewhat lower than that envisioned in McQuade’s mechanism [62]. Experimental support for this dual option-mechanism (Scheme 3) has been recently provided by ESI-MS [63,64,65]. One of the key issues that remain unanswered is the stereochemical outcome of MBH and aza-MBH reactions. In particular, one notes the lack of a unified proposal for the stereochemical outcome of the aldol addition step (step 2) and, in addition, there is uncertainty upon whether or not the original kinetic outcome of the aldol reaction suffers any modification due to reaction reversal driven by thermodynamics (step 3). Needless to say, these issues hamper future development of novel and more efficient catalysts. These stereochemical issues appear to be more complex for the case of MBH reactions where the third step is dual and minor details surely play stereochemically relevant roles that can affect the diastereomeric composition of the so-called aldolate **int2, **and consequently erode the final enantioselectivity. 

**Scheme 4:**
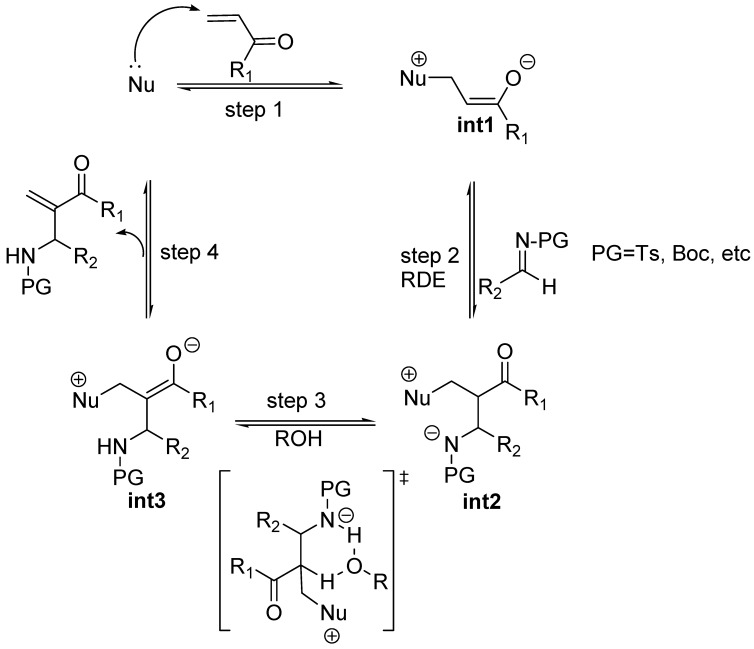
Mechanistic proposal for the aza-MBH reaction co-catalyzed with a Brönsted acid ROH.

In spite of the strong mechanistic analogies between MBH and aza-MBH reactions there are some relevant dissimilarites worth noting here. Both are accelerated by Brönsted acid additives. However, at difference with the McQuade’s findings for MBH reactions, the kinetic studies run by Leitner *et al*. showed that the rate expression for the aza-MBH reaction, carried out in THF, between methyl vinyl ketone with an *N*-tosyl imine in the presence of triphenylphosphine as catalyst, and a Brönsted acid as cocatalyst whose pKa was in the range 16–8, was first order in both imine, ketone and triphenylphosphine, but independent of the acid cocatalyst [66]. The implication was evident: the elimination step of aza-MBH reactions, in which the acid cocatalyst operates, should not be the rate-determining step (Scheme 4). Instead, one could state, provided this behaviour could be extended to all kinds of aza-MBH reactions promoted by bifunctional LABA catalysts, that the rate-determining step should be the Mannich-type addition step also responsible for the eventual stereochemical results, provided racemization of the final product is avoided [66]. The consequence is evident: the race for the development of enantioselective, bifunctional catalysts for MBH and aza-MBH is being won by the latter, no doubt due to the fact that in this case the rate determining step is the addition step (second step) and the third step is fast, which derives in less stereochemical difficulties. 

From a mechanistic point of view there is a second major category of MBH and aza-MBH reactions, namely that which involves the aldol-like addition of an intermediate enamine species, instead of the enolates characteristic of the standard reactions. In particular, secondary α-aminoacid organocatalysts such as (*S*)-proline [67], or (*S*)-pipecolinic acid [68], often assisted with cocatalysts (*N*-methyl imidazole, DABCO, peptides, *etc.*), have recently been added to the above armoury of bifunctional organocatalysts [69,70,71,72]. Obviously, from a mechanistic viewpoint these α-amino acid-organocatalyzed MBH reactions differ completely to those categorized as standard MBH and aza-MBH reactions which involve tertiary amine or phosphine catalysts [73,74,75].

In addition, it is worth mentioning at this point that one can also reach MBH or aza-MBH products by means of non-organocatalytic protocols. These methods either employ Lewis acid catalysts such as BF_3_ or TiCl_4_ with an added halide as cocatalyst [76,77,78,79], or Ti(IV) tetraisopropoxide with, or without a chiral amine [80], phosphine or sulphur compound as co-catalyst [81]. Alternatively, a metallic salt can be used as catalyst or stoichiometric promoter. These miscellaneous methods which employ non-organocatalytic will not be examined in the present minireview.

In spite of the quite large number of reviews dedicated to the standard MBH and aza-MBH reactions no attempt has been made to outline their most prominent stereochemical aspects in a systematic manner, likely because of the still unanswered questions regarding their mechanism. The aim of this minireview is to analyze the stereochemical issues of the two main types of MBH reactions, namely the standard MBH reactions, and the α-aminoacid-cocatalyzed MBH reactions.

## 2. The Standard MBH and Aza-MBH Reactions

For the purpose of examining the stereochemical relevant details of the standard MBH and aza-MBH reactions one needs to consider the general mechanism illustrated in Scheme 3, and keep in mind that those steps taking place before the rate determining step involve reversible reactions and that, according to recent physico-chemical observations, the rate determining step is dual (in other words, it can be one or the other for the same reaction, as a function of time and/or solvent) as a function of the actual experimental conditions, as discussed below. 

It is our contention that standard MBH and aza-MBH reactions should be considered as involving “naked” enolates *i.e.*, fully separated ammonium, or phosphonium, enolates holding specific properties and reactivity [82]. A revealing experiment regarding reversibility of aldol reactions involving fully separated ion pairs (“naked” enolates), was reported in the nineties by Noyori *et al.* for tris(dimethylamino) sulfonium (TAS) enolates. What they found is that TAS enolates do not yield aldol products unless the reaction is driven by an *O*-silylation quench of the aldolate adducts which, accordingly, must be considered high energy species on the reaction profile [83,84]. This behaviour is reminiscent to that of standard MBH and aza-MBH reactions which usually require a protic solvent, a protic cosolvent, or a Lewis Base-Brönsted Acid (LBBA) catalyst for driving the reaction to an efficient level of conversion. 

The first step MBH and aza-MBH reactions (step 1 in Scheme 3) involves the reversible nucleophillic attack upon the β carbon of an α,β-unsaturated system, thereby giving rise, for acyclic systems, to a zwitterionic ammonium or phosphonium, enolate (**int1**) [23,24,25,26,27,28], which can undergo aldol condensation with a C=O or C=N moiety, as reported by Noyori *et al.* for other systems [85,86]. Being this first step reversible, we can expect the formation of a thermodynamically controlled mixture of zwitterionic enolates, the most stable being the *Z*-configured ammonium enolate (thermodynamic enolate) by virtue of the fact that being the O^-^ and CH_2_NR_3_^+^ moieties *cis* to each other [87], strong attractive interactions between the charged oxygen atom and the nearby α-hydrogens of the onium moiety are being developed, as reported by both Houk [88], and Leahy [89]. In this regard it is worth mentioning that Aggarwal, Harvey *et al*. have estimated by means of computation at the B3LYP/6-31+G*/THF level that the thermodynamic (*Z*)-enolate is stabilized by 1,1 kcal/mol relative to the kinetic enolate [87,90]. According to these authors this stabilization of the (*Z*)-enolate is due to the existence of stronger electrostatic interactions in the thermodynamic (*Z*)-enolate, rather than other specific bonding interactions. A recent experimental study has concluded that these specific bonding interactions are not present in the case of a phosphine-catalyzed MBH-like alkylation reaction involving the kinetic zwitterionic phosphonium enolates [91]. The MBH and aza-MBH reaction can thus be appropriately defined as a unique aldol condensation in which the enolate involved is a “naked”, zwitterionic, ammonium, or phosphonium, equilibrating enolate [92,93,94]. Since its formation is highly reversible it can be safely assessed that MBH reactions should involve the above mentioned thermodynamic (*Z*)-enolates as the major species [87]. Curiously enough, the rate of intramolecular MBH reactions has been shown to be highly dependant on the stereochemistry of the Michael acceptor, with the *Z*-stereoisomer being more reactive than the *E-*isomer [95].

**Scheme 5:**
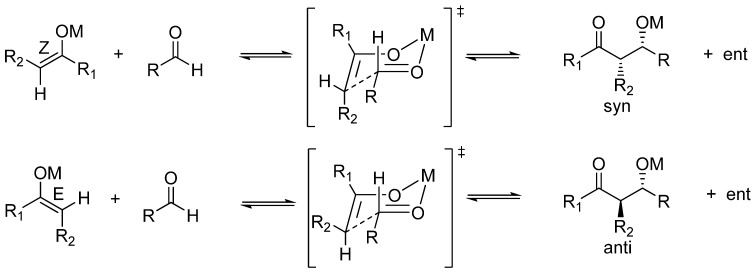
Kinetic diastereoselection for metal enolates according to the Zimmerman-Traxler mechanism of aldol condensations.

Common metal enolates and “naked” enolates are quite dissimilar in reactivity [96,97]. As mentioned previously, the most revealing aspect for the present analysis is that “naked” enolates do not react with aldehydes unless in the presence of trialkylsilyl fluoride which acts as a quencher of the resulting “naked” aldolate, as reported by Noyori *et al*. for TAS enolates [98,99]. A further revealing issue is that of the diastereoselection of aldol condensations. Kinetic diastereoselection when common metal enolates (Li, Na, K, Mg, Zn, B, Ti) are employed is rather well established as a consequence of the running of the so-called Zimmerman-Traxler mechanism which invokes cyclic, chair-like transition states where the metal plays a significant role as it is an integral part of this cyclic array [100]. According to the general ruling for the reactions of these metal enolates, thermodynamic (*Z*)-enolates give rise preferentially to *syn* aldols, whereas (*E*)-enolates lead to *anti* aldols (Scheme 5). 

**Scheme 6:**
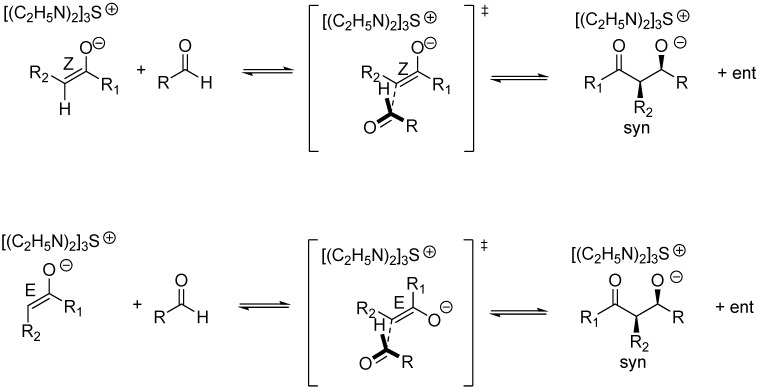
Kinetic diastereoselection of aldol condensations undergone by “naked” enolates as according to Noyori *et al*.

In contrast, a number of aldol reactions do not follow this general rule because a different mechanism is in effect. In particular, as put forward by Noyori and coworkers, “naked” enolates such as TAS enolates give rise to *syn* aldol derivatives regardless of the *E-Z* configuration of the enolate (Scheme 6) [101]. The reason for this is that an acyclic, extended transition state is at work and the energetically most favoured one is that which avoids electrostatic repulsion between the negatively charged oxygens, as illustrated in Scheme 6 [98,99,102,103].

**Scheme 7:**
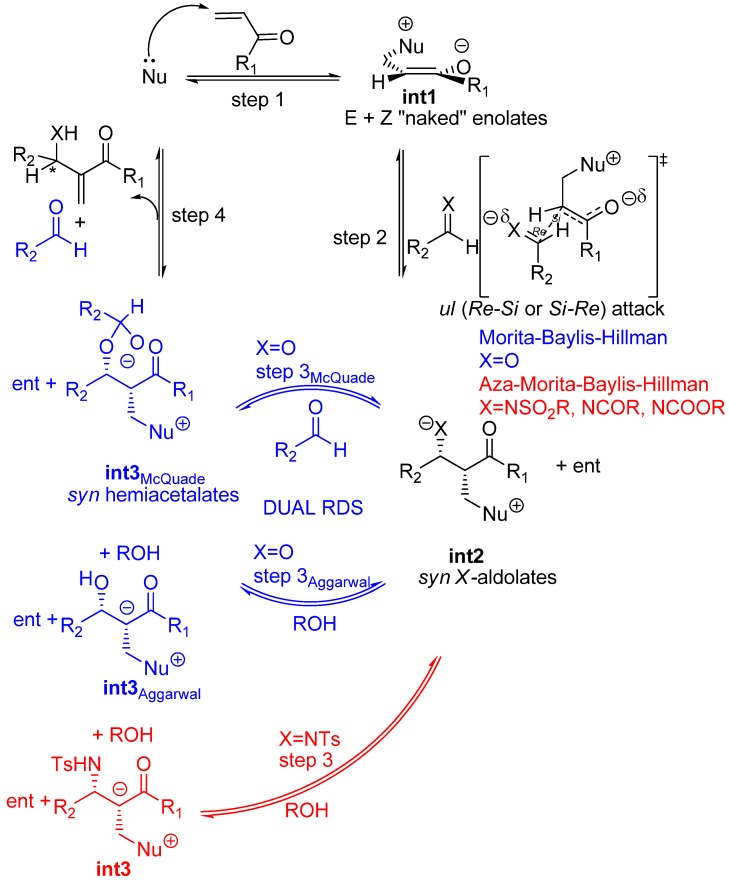
Stereochemical issues for the standard MBH (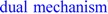) and aza-MBH (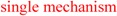) reactions.

Curiously enough, Aggarwal, Harvey *et al*. in their computational study (at the B3LYP/6-31+G*/THF level of theory) of the MBH reaction reported that the lowest transition estate of the C-C bond forming step i.e., that corresponding to the addition of the (*Z*)-configured enolate, was governed by the strength of the dipole-dipole interactions required for maximum electrostatic stabilization [104,105,106], even when an explicit methanol molecule was included for activation [98,99]. Therefore the product stereochemistry should be that of *syn* aldolates, as a consequence of the preferred face selective *ul* (*Re-Si* or *Si-Re*) condensation of either (*Z*)- or (*E*)-configured enolates, as reported by Noyori *et al*. for aldol condensation of TAS “naked” enolates [98,99]. Two additional flag features regarding those aldol reactions undergone by “naked” enolates are worth being mentioned because they perfectly match those of MBH reactions: 1) the final aldolate can react with a second molecule of aldehyde thereby giving rise to dioxane byproducts, and 2) the reaction shows autocatalysis [98,99]. The diastereoselectivity of the addition step to (*E*)-*N*-substituted imines i.e, the rate determining step of aza-MBH reactions, will also be governed by identical principles, though achiral, Brönsted acid additives can actually invert the stereochemical course of the reactions, as shown recently by Masson, Zhu *et al* [107,108]. To sum up, predictions for the standard MBH and aza-MBH reactions are that the ammonium or phosphonium *syn* aldolates should be the kinetic products of the second step (Scheme 7).

**Scheme 8:**
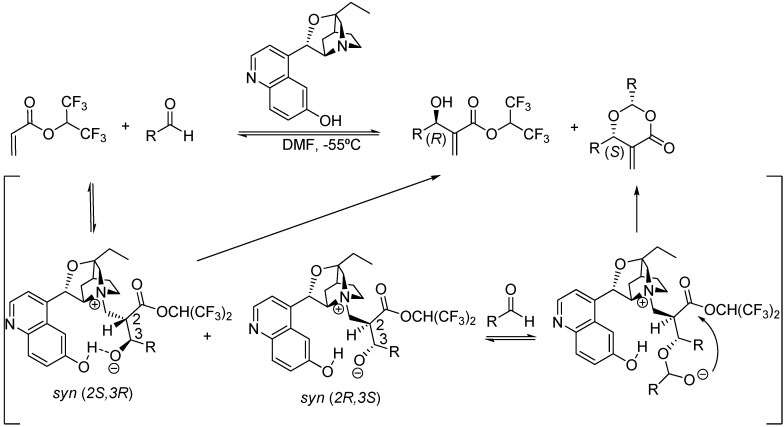
Hatakeyama’s stereochemically divergent β-isocupreidine-catalyzed MBH reaction.

Thus far only scattered information about the intrinsic diastereoselectivity of the MBH and aza-MBH reactions had been advanced in the published literature due to the nature of the third step in which one of the two centers of chirality is eventually destroyed. A particular case is the mechanistic rationale proposed by Hatakeyama *et al*. for explaining the intriguing opposite enantioselectivity observed for the common MBH product and for the unexpected dioxanone adduct obtained in the β-isocupreidine-catalyzed MBH reaction between 1,1,1,3,3,3-hexafluoroisopropyl acrylate with aliphatic or aromatic aldehydes in DMF at -55 °C [109]. The authors called for the formation of the two diastereoisomeric *syn* adducts that evolved, presumably at similar rates, to the divergent products. In particular, the *syn* (*2S,3R*) adduct undergoes direct β-elimination thereby yielding the (*R*)-MBH product, whereas the *syn* (*2R,3S*) reacted with a second aldehyde molecule, as predicted by the McQuade’s dual mechanism, thereby giving rise to the (*S*)-dioxanone byproduct which has the opposite configuration, as shown in Scheme 8. 

**Scheme 9:**
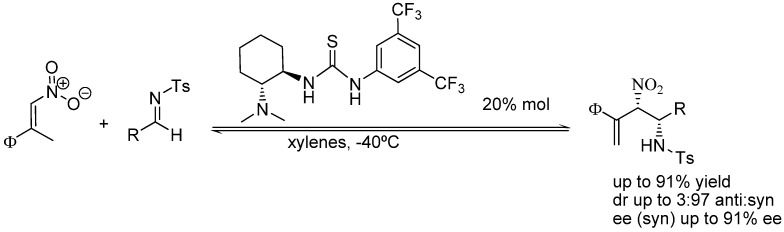
Diastereo and enantioselective aza-MBH reaction of nitroalkenes with *N*-Ts protected imines catalyzed by bifunctional amino thioureas.

Fortunately, a recent work by Xu *et al*. has provided clear-cut proof for the intrinsic diastereoselectivity, as well as the enantioselectivity, of the aza-MBH reaction of nitroalkenes with *N*-tosylimines catalyzed by several chiral amino thioureas derived either from quinine or 1,2-trans-diaminociclohexanes [110], which supports the above analysis. Instead of the prototypical proton shift and β-elimination, this reaction evolved through direct β-elimination thus resulting in the formation β-nitro-γ-enamines containing the two contiguous centers of chirality previously generated in the addition step. The results shown in Scheme 9 for the reaction catalyzed by (1*R*,2*R*)-diaminocyclohexane thiourea clearly provide sound support of the above reasoning as all reactions run in apolar, aprotic media yielded *syn* β-nitro-γ-enamines in high diastereomeric ratio, whereas those carried out in polar and protic solvents led to much lower diastereoselection.

Nonetheless, a cautionary note is needed. Even though Noyori reported that the *syn* or *antiO*-silylated aldols are configurationally stable in the presence of fluoride in an aprotic medium, one should not forget that stereochemically defined lithium aldolates can suffer stereochemical isomerization in protic media [111]. Accordingly, at the time of devising new enantioselective catalysts for the MBH reaction, best candidates ought to be those that allow for a rapid protonation of the *syn* aldolates (**int2**) formed in the second step of MBH aldol condensations. In other words, the role of a bifunctional LBBA catalyst may be that of providing for a rapid protonation of the *syn* aldolate (**int2**) thereby giving rise to a basic site appropriately located for promoting a rapid β-elimination, *i.e*, promoting a concerted protonation-deprotonation sequence. Recent theoretical studies have provided evidences for declaring the second step the rate determining step for MBH reactions carried out in the presence of protic solvents or dual Lewis base-Brönsted acid catalysts [112,113,114], or the final β-elimination when in the absence of protic solvents or catalysts [56,60,115]. Trifunctional or multifunctional catalysts are conceivable and, in fact, some has already been described [116,117].

The lesson to be learned for the design of catalysts is clear: bifunctional LBBA molecules appear to be useful candidates for enantioselective catalysts for the standard MBH and aza-MBH reactions. Obviously, not all bifunctional LBBA catalysts will work, and in fact many of the so-called privileged catalysts have failed. In our view, in order to reach efficiency, catalysts will need to consider the spatial disposition of the dipoles in the stereochemically relevant transition state, as recently shown by Clarke, Philp *et al.* [118].

**Scheme 10:**
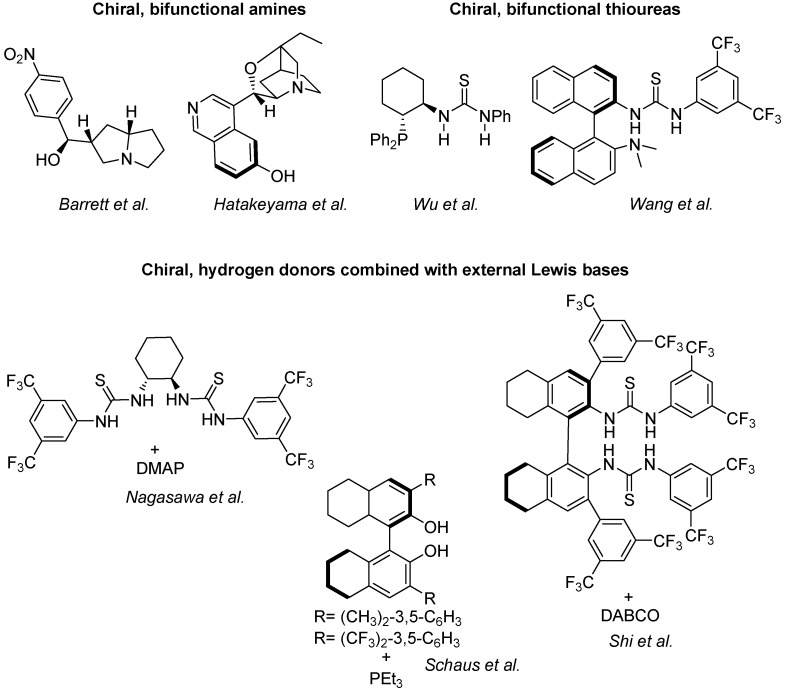
Catalytic systems for the standard, enantioselective MBH reactions.

Quite a large armoury of highly successful enantioselective catalysts for standard MBH reactions is already available. Some of them, illustrated in Scheme 10, are classified in three main groups, namely the chiral bifunctional amines of Barrett [52] and Hatakeyama [109], the chiral, bifunctional thioureas of Wu [119] and Wang [120], and the chiral hydrogen donors combined with Lewis bases of Nagasawa [121], Schaus [122] and Shi [123].

**Scheme 11:**
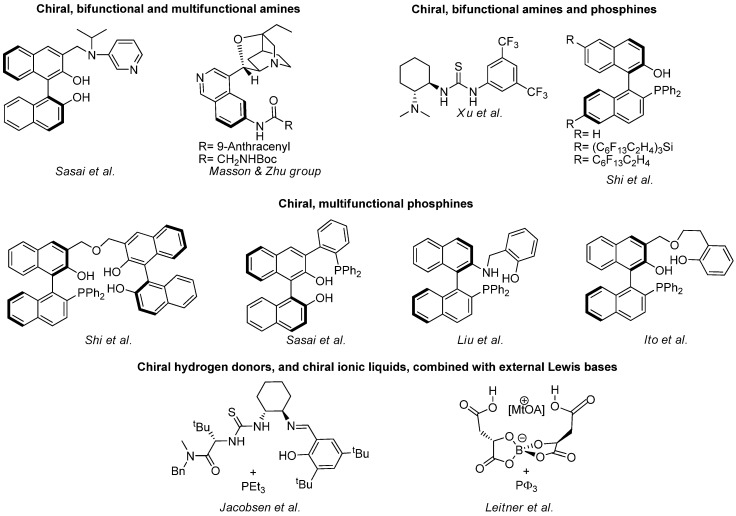
Enantioselective catalysts for the standard aza-MBH reaction.

The number of enantioselective catalysts for the aza-MBH reaction has grown exponentially in recent years. Some of them, illustrated in Scheme 11, can be classified in four main groups, namely the chiral bifunctional and multifunctional amines of Sasai [124,125], and Mason and Zu [107,108], the chiral bifunctional amines and phosphines of Xu [110] and Shi [126,127,128,42], the chiral multifunctional phosphines of Shi [129,130], Sasai [58], Liu [116,117], and Ito [131], and the chiral hydrogen donors, as well as chiral ionic liquids, combined with external Lewis base of Jacobsen [44], and Leitner *et al.* [156].

## 3. The α-Aminoacid Catalyzed and α-Aminoacid-Amine Cocatalyzed Mbh and Aza-Mbh Reactions

Secondary amines were recognized to promote intramolecular cyclizations of enonealdehydes thereby yielding MBH products. The reaction was interpreted, however, as involving a tandem Michael/aldol condensation [132,133,134]. To the best of our knowledge the first reported attempt at employing a secondary amine such as (*S*)-proline as enantioselective catalyst for MBH reactions is due to Shi and coworkers [135]. Their discovery was quite simple but fundamental: even though (*S*)-proline itself failed to promote the MBH reaction of arylaldehydes with a β-unsubstituted α,β-unsaturated ketone such as methyl vinyl ketone (MVK), the presence of an equimolar amount of a Lewis base such as imidazole, benzimidazole or DABCO acting as co-catalysts led to the corresponding MBH adducts in high yield, though in very low enantioselectivity (5–10% ee), the use of such chiral tertiary amines as Hatakeyama’s β-isocupreidine leading only to a somewhat marginal improvement in enantioselectivity [136].

**Scheme 12:**
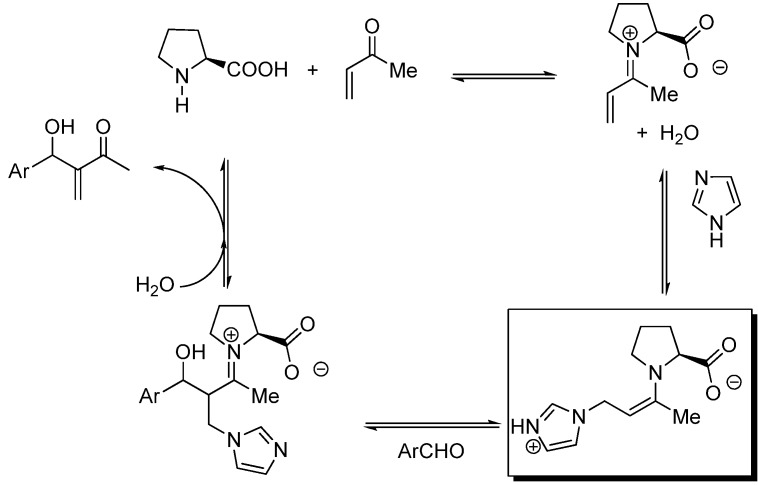
General mechanism for the MBH (*S*)-proline-Lewis base co-catalyzed reactions.

**Scheme 13:**
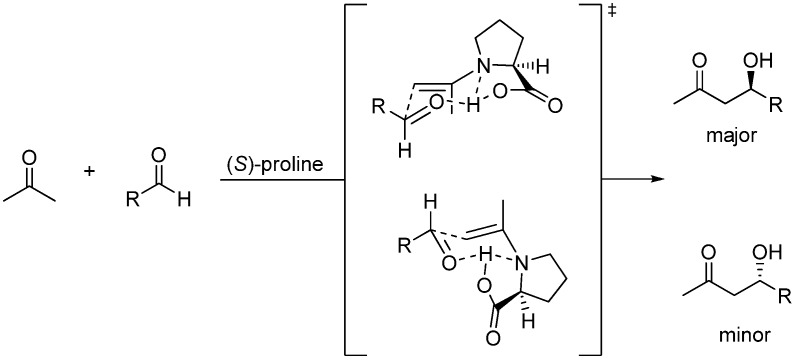
Generalized mechanism for (*S*)-proline-catalyzed aldol reactions based on the Zimmerman-Traxler model.

Inspired by the (*S*)-proline organocatalyzed aldol condensations reported by List [137,138], Barbas [139], and MacMillan [140], Shi *et al*. proposed a mechanism (Scheme 12) involving formation of an intermediate enamine species (highlighted in Scheme 12) derived from the trapping of the conjugated iminium species resulting from the condensation of (*S*)-proline with MVK by the co-catalyst which bears a clear-cut relationship with the zwitterionic enolates (**int1**) of the standard MBH reactions previously mentioned. The stereochemical issues regarding (*S*)-proline-catalized intermolecular aldol additions have been well established by Houk, List and coworkers [141]. Accordingly, Shi’s enamine intermediate should react with the aldehyde in keeping with the generalized mechanism based on the Zimmerman-Traxler six-membered ring chair-like model [100]. Eventually, recycling of the catalyst should take place as a consequence of hydrolysis of the final iminium condensate (Scheme 13). At the time of writting this review it is clear that there is much room for improvement of the enantioselectivity of MBH co-catalyzed reactions.

Other chiral, tertiary amino alcohols have also been explored for synergy with (*S*)-proline in catalyzing enantioselective MBH reactions by Zhou, He and coworkers with some improvement [142,143]. Miller’s approach to reach ideal synergy called for exploring π-(Me)His (Pmh)-containing peptides of various lengths and constitution [144]. The best enantioselection (78% ee) for the prototypical reaction of the *o*-nitrobenzaldehyde with an β-unsubstituted α,β-unsaturated (MVK) was obtained when using (*S*)-proline with the chiral octapeptide BOC-(π-Me)His-Aib-Chg-(trt)Gln-D-Phe-D-Pip-Cha-Phe-OMe as cocatalyst, the key observation being that the “unmatched” pair (*R*)-proline/ BOC-(π-Me)His-Aib-Chg-(trt)Gln-D-Phe-D-Pip-Cha-Phe-OMe yielded the MBH adduct with opposite configuration in only 33% ee. This observation led the authors to conclude that the stereochemical issues regarding proline/tertiary amine cocatalyzed MBH reactions are dominated by proline stereochemistry. The scope of the reaction has been examined [145].

Application of these ideas to an intramolecular MBH cyclization led Miller and coworkers to find that the (*S*)-pipecolinic acid/N-methylimidazole pair gave better enantioselectivities than the (*S*)-proline/N-methylimidazole pair [146]. Simultaneously, Hong and coworkers not only observed that (*S*)-proline itself behaved as an effective catalyst but also reported the unexpected observation that the Lewis base co-catalyst employed (e.g., imidazole) gave rise to inversion of the enantioselectivity, an event that required a new reactive intermediate, as suggested by the authors [147]. From a mechanistic viewpoint, it was proposed that the reaction catalyzed by (*S*)-proline involved (*E,E*)-dienamine species which followed the widely accepted enamine mechanism as applied to an intramolecular aldol condensation, whereas that co-catalyzed by imidazole involved imidazole-substituted (*E*)-enamines having two centers of chirality [148,149]. 

**Scheme 14:**
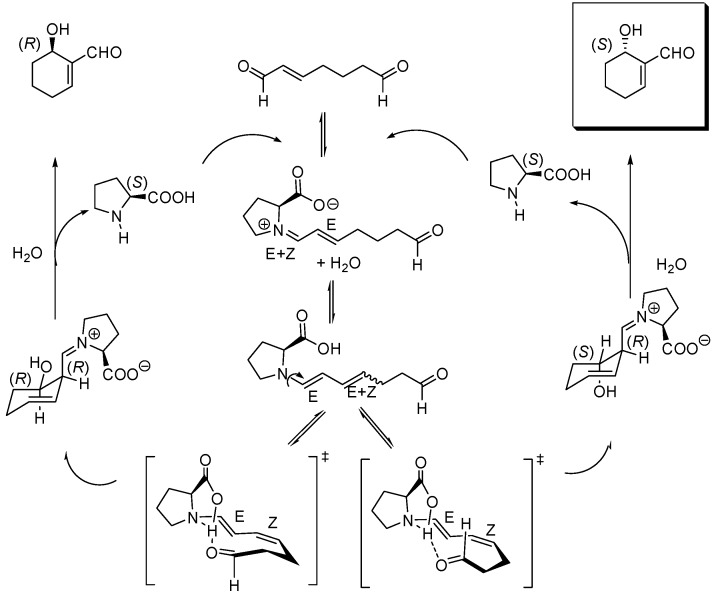
Mechanism for the (*S*)-proline-catalyzed intramolecular cyclization of hept-2-enedial to (*S*)-6-hydroxy-cyclohex-1-enecarbaldehyde.

Subtle details of this mechanism have come to light as a consequence of an extensive density functional analysis at the B3LYP/6-31-G(d,p) level which included the use of the polarized continuum model (PCM B3LYP/6-31++G(d,p)//B3LYP/6-31G(d,p)) to describe solvent effects, the most important being the role played by water to give rise to the required *syn* and *anti*, (*E,Z*)-dienamine key intermediates in equilibrium, as the theoretical calculations demonstrated that (*E,E*)-enamines could not undergo cyclization [150]. According to these PCM calculations, the cyclization of the *anti*, *E,Z*-dienamine is biased towards the formation of the (*S*)-configured product, as illustrated in Scheme 14 which displays, in a simplified manner, the modifications introduced by the computational work by Gil Santos *et al*., in close analogy with the model proposed and by List and coworkers [151].

**Scheme 15:**
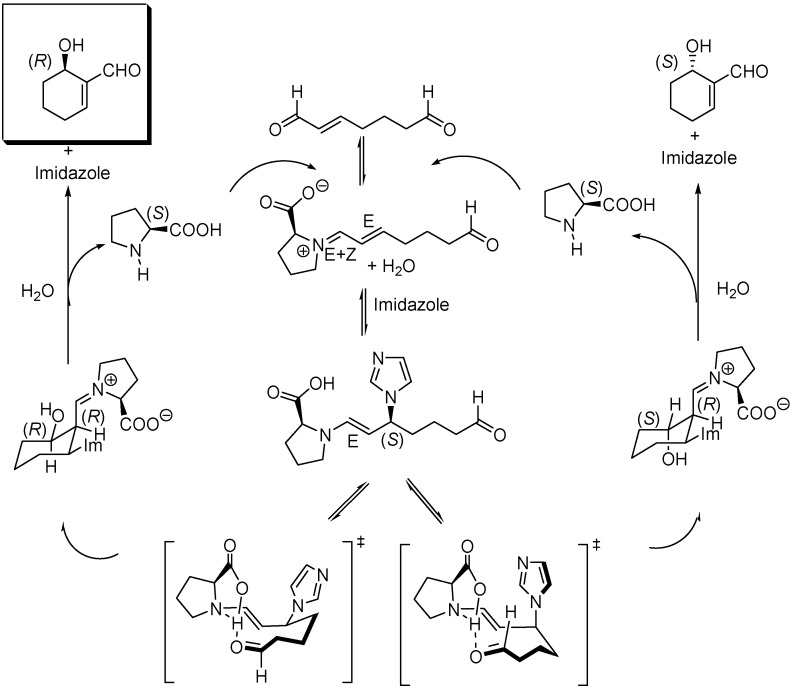
Mechanism for the (*S*)-proline, imidazole co-catalyzed intramolecular cyclization of hept-2-enedial to (*R*)-6-hydroxycyclohex-1-enecarbaldehyde.

The original mechanistic proposal of Hong *et al.* for the imidazole co-catalyzed reaction has also been modified as a consequence of the theoretical studies of Gil Santos *et al*., which predict the formation of the (*S,S*)-diastereoisomer of the 3-(1-imidazolyl)-substituted enamine by attack of imidazole to the (*E*)-iminium ion assisted by water in the rate limiting step. Cyclization of this intermediate followed by hydrolysis yields the (*R*)-6-hydroxycyclohex-1-enecarbaldehyde and (*S*)-proline, as illustrated in Scheme 15. Calculations also provide satisfactory data for explaining the temperature and solvent polarity dependence of the cyclization. 

**Scheme 16:**
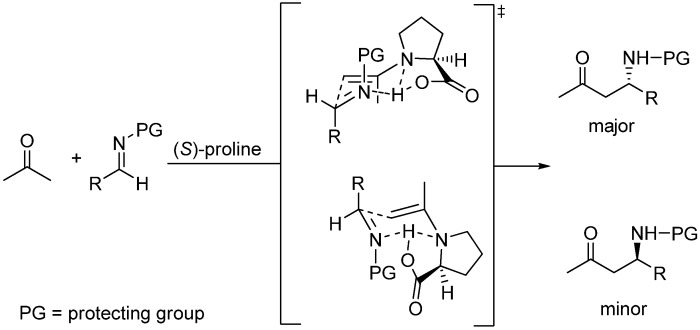
Generalized mechanism for (*S*)-proline-catalyzed Mannich reactions based on the Zimmerman-Traxler model.

First examples of aza-MBH reactions catalyzed by (*S*)-proline, as well as co-catalyzed with added bases, have come to light quite recently first published by Barbas, Tanaka and coworkers [152], and then by Córdova and coworkers [153]. From a stereochemical viewpoint, intermolecular reactions of β-alkyl substituted-α,β-unsaturated aldehydes with either *N*-PMP (Barbas and Tanaka) or *N*-BOC protected imines (Córdova) catalyzed by (*S*)-proline, can be understood as Mannich condensations involving either a (*E,E*)-dienamine, or the 3-substituted enamine resulting from the trapping of the precursory conjugated iminium ion by the cocatalyst. Whichever the case, a Zimmerman-Traxler six-membered ring chair-like model as applied to N-substituted imines (Scheme 16) should apply [154,155].

**Scheme 17:**
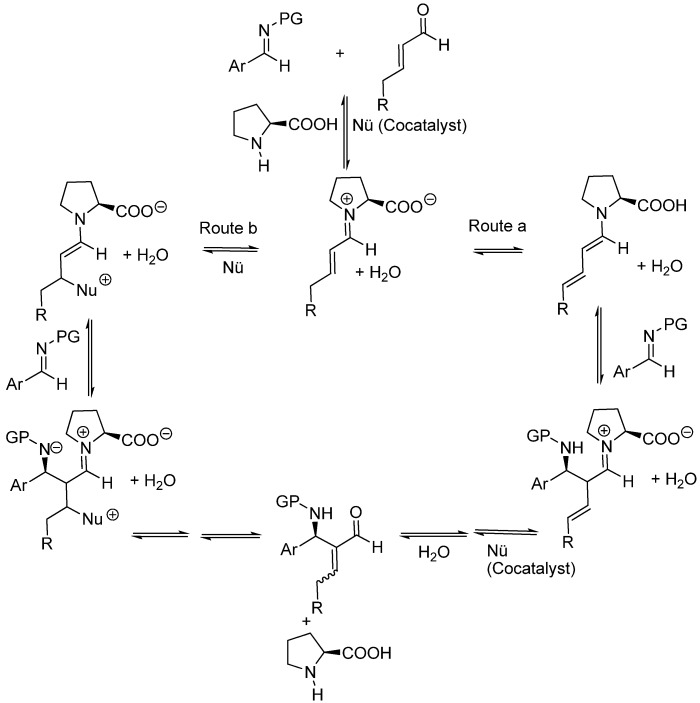
Alternative mechanisms for (*S*)-proline-amine co-catalyzed Mannich reactions of β-substituted aldehydes and *N*-protected imines (*N*-PG).

The available evidence found by Barbas, Tanaka and coworkers suggests that the role of the co-catalyst (imidazole) was only that of increasing the rate of the reaction and improving the chemical yield, but had no influence on the enantioselectivity and absolute configuration of the final adducts, therefore implying that the reaction actually involves the condensation of a (*E,E*)-dienamine with the protected imine (route a in Scheme 17). Córdova and coworkers did not find positive evidences for distinguishing between the dienamine route (route a) and the 3-substituted enamine route (route b).

## 4. Conclusions

Major efforts are being dedicated to the search of chemically efficient, enantioselective, organocatalytic Morita-Baylis-Hillman (MBH) and aza-Morita-Baylis-Hillman (aza-MBH) reactions. These reactions provide enantiomerically-enriched, densely-functionalized molecules of interest to synthetic organic chemists. The development of efficient reactions has been plagued with difficulties derived from low conversions, meagre chemical yields and poor enantioselectivities. Fortunately, along the years, chemists in their search for better oganocatalysts have identified a number of key mechanistic issues by means of fundamental, physical organic chemistry studies, and otherwise. Breakthroughs focussing on the nature of the rate determining step of both the MBH and aza-MBH reactions, and of course those that reveal the nature of the species actually involved paved the way for the actual development of the two major avenues that lead to efficient, enantioselective, organocatalytic MBH and aza-MBH methodologies. 

The so-called standard MBH and aza-MBH reactions involve Lewis base catalysts (typically tertiary amines or phosphines). Efficient catalytic systems for them either entail an external Lewis base and a chiral hydrogen donor, or instead the catalyst usually is a single chiral, bifunctional or multifunctional molecule having both a Lewis base and one or several hydrogen donors appropriately located in space. However, we believe that there is still room for improvement. This minireview has examined the stereochemical issues regarding these reactions in a coherent manner. The most relevant conclusion of this analysis is that standard MBH and aza-MBH reactions involve the aldol-type condensation of “naked” enolates, thereby leading to *syn* adducts irrespective of the configuration of the enolate. Several pieces of evidence already available in the published literature support this conclusion. Accordingly, provided the second step is rate determining step, the design of successful bifunctional or polyfunctional catalysts has to consider the geometrical requirements imposed by the transition structures of the second step of these reactions. 

On the other hand, all MBH and aza-MBH reactions promoted by both (*S*)-proline and a co-catalyst (a secondary or tertiary amine) invoke the aldol-type condensation of either a 3-amino substituted enamine, dienamine, or both, depending on cases. The stereochemical issues regarding these co-catalyzed condensations appear to mirror those of the well established (*S*)-proline catalyzed aldol-like reactions.
